# Validation of Vitamin D-Specific Food Frequency Questionnaire against Food Records for Qatari Women

**DOI:** 10.3390/foods9020195

**Published:** 2020-02-14

**Authors:** Vijay Ganji, Reem Abu-Dbaa, Haneen Othman, Menatallah Zewein, Tamara Al-Abdi, Zumin Shi

**Affiliations:** Human Nutrition Department, College of Health Sciences, QU Health, Qatar University, P.O. Box 2713, Doha, Qatar; ra1402952@student.qu.edu.qa (R.A.-D.); ho1406467@student.qu.edu.qa (H.O.); mz1404717@student.qu.edu.qa (M.Z.); tamara.alabdi@qu.edu.qa (T.A.-A.); zumin@qu.edu.qa (Z.S.)

**Keywords:** FFQ, food frequency questionnaire, food records, Qatar, validation, vitamin D

## Abstract

The measurement of vitamin D nutritional status through dietary assessment is cost effective. Food frequency questionnaire (FFQ) is usually validated against food records (FR). There is no vitamin D-specific FFQ for Qatar population. The objective of this study was to develop a vitamin D-centric FFQ and validate FFQ against three-day FR for Qatar population. A quantitative FFQ based on vitamin D containing foods consumed in Qatar was developed. Vitamin D contents of foods were gathered from food labels and food composition tables from the United States Department of Agriculture. A vitamin D content database was developed for this study purpose. Dietary intakes while using FFQ and three-day FR were collected from 62 women. Vitamin D intakes from FFQ and three-day FR were validated with quartile comparison and Bland-Altman (BA) tests. BA plot showed an agreement between FFQ and three-day FR vitamin D intakes. The BA index was 3.23%, which is <5%, a commonly used standard for validation. Quartile correlation showed that ≈73% of subjects were within the same or adjacent quartile. In conclusion, an agreement was found between vitamin D intakes from FFQ and three-day FR in Qatari women. More studies are needed to validate the vitamin D-specific FFQ in Qatari population at large.

## 1. Introduction

Vitamin D is a lipophilic nutrient that was obtained from the diet and vitamin D supplements. Although vitamin D is synthesized endogenously in the skin when exposed to Sun’s UV-B light, the amount of vitamin D that is synthesized in this process is highly variable and affected by several factors, such as latitude, time of the day, skin tone, amount of clothing worn, and use of sunblock lotions [1,2,3]. Interest in vitamin D has grown in recent times because hypovitaminosis D has been found to be related to non-calcemic ailments, such as metabolic syndrome [4,5] obesity [6], diabetes mellitus [7], cancer [8,9], cardiovascular diseases [10], and depression [11]. However, the cause and effect relationship between vitamin D and chronic diseases is still under investigation.

Vitamin D insufficiency has been a worldwide health problem [12]. Approximately 90% of the population in Qatar suffers from vitamin D deficiency or insufficiency [5]. The dietary sources of vitamin D are limited. These are egg yolks, oily fish, fortified dairy, beef liver, and ready-to-eat breakfast cereals [13]. The diet becomes an important source of vitamin D because avoidance of exposure to sunlight is common in countries like Qatar due to extreme heat [14]. Vitamin D status can be measured while using serum 25-hydroxyvitamin D [25(OH)D] concentrations, however this biochemical assessment of vitamin D is invasive and expensive [15].

The dietary assessment methods are cost effective and are suitable for screening of nutrient intakes at a population level [16]. Among all of the dietary assessment methods, the Food frequency questionnaire (FFQ) is a widely used tool in estimating the nutrient intakes of populations [17]. The FFQ captures habitual dietary intakes of populations better than food records (FR) or 24-hour recalls [18]. Moreover, the FFQ is a widely used dietary assessment method, because it captures the long-term dietary intake of populations [18] and has a less respondent burden [19]. Previously several investigators validated FFQ in various populations [19,20]. Steinemann et al. [19] reported that FFQ had reasonable relative validity for various food groups, such as fruits, nuts, meat, sausage, eggs, salty snacks, and beverages. Araujo et al. [20] reported an agreement between semiquantitative FFQ and FR for energy and nutrient intakes.

Before a specific dietary assessment tool for collecting the habitual intake of population is used, it must be validated against a reference standard. Validation is a process of verifying whether a certain type of assessment method would be useful as a measure or indicator for providing useful analytical measurements for a specific purpose and context [21]. FFQs are commonly validated against food records (FR) [19,20,22]. We used FR as a reference standard for FFQ validation because no gold standard exists. It is important to develop and validate a FFQ to measure the vitamin D status of the Qatar population because there is no vitamin D-specific FFQ available. Therefore, the objective of this study was to develop a vitamin D-specific FFQ and validate this FFQ against FR for the population in Qatar.

## 2. Materials and Methods

### 2.1. Development of Vitamin D Content Database for Qatari Foods

Vitamin D containing foods were collected by visiting the main grocery stores (Al Meera, Carrefour, and Lulu supermarkets) in the city of Doha, Qatar. The vitamin D contents of foods were gathered from the nutrition label on the package or container. However, the United States Department of Agriculture (USDA) Food Composition database was used to calculate the vitamin D content for those foods with no vitamin D content on the nutrition label. For example, the vitamin D content of beef, liver, and fish was not available, because they were sold fresh. Recipes were collected from the Gulf Council Cooperation (GCC) food composition tables in order to calculate vitamin D content of mixed ingredient foods. In addition, the recipes were gathered from cook pads and websites specialized for Qatari food recipes. Only recipes with vitamin D containing ingredients (eggs, fish, dairy, and fortified cereals) were then collected. The vitamin D content of the recipes was calculated based on the vitamin D content of individual ingredients and the amount used in those recipes.

### 2.2. Development of Vitamin D-Specific Quantitative FFQ

Vitamin D-specific quantitative FFQ was based on four main food items. These food items were eggs, dairy, fish, and fortified cereals. The subjects were asked to provide the frequency of foods (never, daily, weekly, monthly, and yearly) consumed in the last year. Additionally, the serving sizes were listed for each food item on the FFQ. The serving sizes were based on what is usually consumed by the Qatar population. The subjects were provided a serving size guide for accurate estimation of quantity of food consumed. Brand names were also collected whenever applicable. In addition to dietary intakes, we collected demographic information, such as age, education, nationality, and socioeconomic status. Additionally, data on lactose intolerance, food allergies, diet restriction (veganism or vegetarianism), and chronic diseases were collected. Dietary intakes were collected through face-to-face interaction. 

### 2.3. Three-Day FR

The subjects were asked to provide a three-day FR for two consecutive weekdays and one weekend day. Subjects received blank three-day FR sheets with instructions on how to record the food intakes as well as the guide on portion sizes. The subjects were instructed to choose those days that represented their typical intake on those days. Subjects returned completed three-day FR, either electronically or in person.

### 2.4. Dietary Intake Assessment for Validation of FFQ against Three-Day FR

The Institutional Review Board (IRB) of Qatar University approved this study for the involvement of human subjects. The subjects signed a written consent form before their participation. The inclusion criteria of this study consisted of at least 18 years of age, have background knowledge regarding serving size, and have lived in Qatar for two or more years. The subjects should be nutrition students in the Human Nutrition Department at Qatar University. Nutrition students were selected, because they are familiar with the serving size, as well as the procedures to fill out FFQ and FR. On the other hand, exclusion criteria were those who were pregnant, those with lactose intolerance, those with no vitamin D intake either from FFQ or three-day FR, and those following a specific diet, such as veganism or vegetarianism.

A total of 93 subjects were recruited for the validation of FFQ against FR. However, four of them were excluded from the study because of pregnancy (*n* = 1), veganisam (*n* = 1), and lactose intolerance (*n* = 2). Out of the 89 subjects eligible for the study, 63 subjects completed FFQ and returned three-day FR. One subject’s data were excluded from the analysis due to a lack of reported vitamin D intake. The final validation analysis included dietary intake records from 62 subjects.

### 2.5. Determination of Vitamin D Intake from FFQ and FR

Vitamin D intakes from FFQ and three-day FR were determined based on the vitamin D composition database that was developed for the purpose of this study. Quantitative FFQ data were converted to daily vitamin D intake. For example, if a subject recorded that she had eaten a food two times per week (each time = 1 serving), the total vitamin D intake was calculated based on the vitamin D content of that food, times two, and divided by seven to get the daily vitamin D intake for that food. Similarly, for a monthly frequency, the vitamin D intake was divided by 30 and for yearly frequency, the vitamin D intake was divided by 365 to obtain the daily vitamin D intake. All of the vitamin D contents from all foods by each subject were summed to obtain daily vitamin D intake per subject.

Vitamin D intake from all foods reported in a three-day FR were computed while using the same vitamin D database that was developed for this study. Vitamin D intake for each subject, for each food item, for each day was computed. Subsequently, an average of the three days of vitamin D intake was calculated per subject. The vitamin D intake from supplements was not considered in the daily vitamin D intake.

### 2.6. Statistical Analysis

Dietary vitamin D intake data from FFQ and three-day FR were tested for normality. Based on the Shipiro-Wilk test, vitamin D intake data from FFQ and three-day FR were not normal. Therefore, the vitamin D intake data were presented as median and inter quartile range. We used the Spearman’s Rank Correlation to study the relation between vitamin D intakes from FFQ and three-day FR because the data were not normal. We defined the correlation coefficients as very well correlated (0.7 to 0.9), well correlated (0.5 to 0.7), and moderately well correlated (0.3 to 0.5) [23,24]. We have performed the regression between vitamin D intakes of FFQ and FR. Additionally, the vitamin D intake data between FFQ and FR were compared with the Wilcoxon-Signed-Rank test.

The Bland-Altman (BA) plot was used to determine the agreement between vitamin D intake data from FFQ and three-day FR. The limits of agreement (LOA) values were established as the mean difference of dietary intake of vitamin D measured from FFQ and three-day FR ± standard deviation of the difference of vitamin D intake of two methods multiplied by 1.96 (mean difference ± 1.96 × SD). The differences in vitamin D intake between FFQ and three-day FR were plotted against the mean of FFQ and three-day FR vitamin D intakes. If the 95% of the differences (vitamin D intake difference between FFQ and FR) fall within the 95% LOA, then it is considered to be a good agreement. A BA index of less than 5% means that 95% of the vitamin D intakes lie within the LOA. We calculated the BA index based on how many differences lie outside the LOA (the number of differences outside the LOA/total number of differences × 100) [25]. 

Furthermore, the agreement between vitamin D intake data from the two methods were determined while using the cross-quartile classification analysis to validate the agreement between the vitamin D intakes of FFQ and FR methods. First, the vitamin D intakes from the FFQ were classified into quartiles and compared with the quartiles of vitamin D intakes from the FR. The proportion and number of subjects for vitamin D intake from FFQ and FR were classified into same quartile (vitamin D intake quartiles (n) from FFQ match (n), with the intake quartiles of FR (n)), adjacent quartile (±1 quartile), distant quartiles (two quartiles apart), and opposite quartiles (misclassified) [22]. We used a criterion of more than 2/3^rd^ of the vitamin D intakes were in the same or ±1 quartile to define the agreement between vitamin D intakes from FFQ and FR. Additionally, we have calculated Cohen’s weighted kappa (κ) value to assess the extent of agreement. We used the cut-off points proposed by Flight et al. to interpret the κ values [26]. A κ value of less than 0.20 indicated poor agreement, 0.21–0.40 indicated fair agreement, 0.41–0.60 indicated moderate agreement, 0.61–0.80 indicated good agreement, and 0.81–1.0 indicated very good agreement. In all data analyses, a *p*-value of <0.05 was considered to be statistically significant. All of the analyses were performed with STATA (Stata, College Station, TX, USA).

## 3. Results

All of the study participants in the validation study were women. They were in the age group of 19 to 22 years. The survey response rate was ≈71% (63 out of 89). They were all enrolled in the human nutrition major at Qatar University at the time of the study. The median (interquartile range) vitamin D intake per day from the FFQ was 239.2 IU (259.3 IU) and from the FR was 111.3 IU (125.6 IU). The Spearman’s Rank Correlation coefficient between the vitamin D intake from FFQ and FR was significant (rho, 0.36; *p* < 0.005), suggesting a moderate association between vitamin D intakes of FFQ and FR. The regression analysis showed a weak relationship between vitamin D intakes of FFQ and FR (*r*^2^ = 0.13), although the regression coefficient is significant (*p* = 0.004). However, the mean intakes of vitamin D between FFQ and FR was significant (*p* < 0.05). The mean dietary intakes of vitamin D from FFQ and FR were 294.8 IU and 142.7 IU, respectively. The FFQ has a bias of 152.1 IU (the mean difference). Therefore, the FFQ overestimated the vitamin D intakes by 152.1 IU as compared to the FR. 

**Figure 1:**
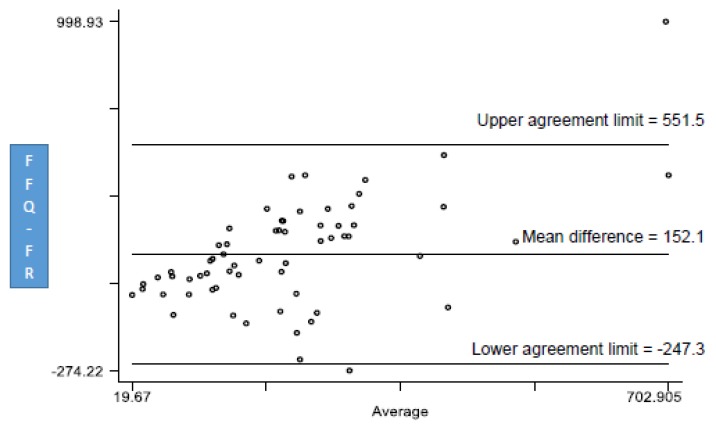
The Bland-Altman (BA) plot showing “differences in mean daily vitamin D intake between food frequency questionnaire (FFQ) and three-day food records (three-day FR)’ against the average daily vitamin D intake of FFQ and three-day FR”. Only two out of 62 fell out of lower and upper agreement limits, giving a BA index of ≈3.2%.

Figure 1 presents the BA agreement plot. There was an agreement between vitamin D intake from FFQ and vitamin D intake from FR. The mean difference between the vitamin D intake from FFQ and from three-day FR was 152.1 IU. The upper agreement limit was 551.5 and the lower agreement limit was -247.3. The majority of the vitamin D intake values were within these agreement limits. Only two vitamin D intake values were out of the agreement limits, giving a BA index of ≈3.2% (2/62 × 100). Approximately 97% (60 out of 62) vitamin D intakes were within the agreement limits.

Table 1 presents quartile comparisons between the vitamin D intakes from FFQ and three-day FR. Out of 62, 23 (≈37%) subjects’ vitamin D intake estimated from FFQ were categorized into the same quartile vitamin D intake as FR. Out of 62, 45 (≈73%) subject’s vitamin D intake from FFQ were categorized into the same or adjacent (± 1) quartile vitamin D intake as FR. Only four out 62 (≈6%) participants’ vitamin D intakes were placed in the opposite quartile (misclassified). The quartile classification of vitamin D intakes from FFQ and FR tools indicates good agreement between these two dietary assessments.

The weighted κ value was 0.3, which showed fair agreement between vitamin D intakes of FFQ and FR. The κ measures the level of agreement between two measurements on the same participant. In cross-quartile analysis, there were four categories, so we calculated weighted κ. Cohen κ is an extension of cross-quartile classification.

## 4. Discussion

The objective of the study was to develop a vitamin D-specific FFQ and validate the vitamin D intake from FFQ against the three-day FR. The validation of FFQ against a reference standard was necessary to determine an agreement between the FFQ and a reference because there is no vitamin D-specific FFQ for Qatar’s population. The reference standard that is commonly used in dietary validation studies is FR. Based on the BA agreement plot, there was an agreement between the vitamin D intakes from the FFQ and the three-day FR. In the BA plot, all of the vitamin D intakes of subjects, except two were positioned between the upper and lower agreement level. The BA index of 5% is used as a cutoff point for determination of agreement between two methods. The lower the BA index, the greater the agreement between the two methods [27]. A BA index of 3.23% was obtained in this study. Similarly, a study by Park et al. [28] assessed calcium and vitamin D intake of Korean women by comparing two specific FFQs, i.e., the Korean calcium assessment tool (KCAT) and the Canadian calcium assessment tool (CAT). Park et al. [28] obtained a BA index of 3.1% for women under the age of 50 years and 3.9% in the general group indicating an agreement between KCAT and CAT. Faid et al. [29] assessed vitamin D intake in Libyan women. In their validation analysis, they attained an index of 5.1% between FFQ and FR. A study by Pritchard et al. [30] was conducted to validate the FFQ against a five-day FR to assess vitamin D, calcium, and vitamin K intakes in overweight and obese post-menopausal women. They obtained a BA index of 6.7%. Similarly, a study that was conducted by Taylor et al. [31] validated FFQ against a four-day FR for the assessment of vitamin D and calcium in teenage anorexic women and obtained an index of 6.3%. The results by Faid et al. [29], Pritchard et al. [30], and Taylor et al. [31] obtained greater BA indices than our study, which indicates lesser agreement than our study.

Although the BA plot indicated an agreement between vitamin D intakes of FFQ and three-day FR (intakes of subjects are within the agreement limits), the vitamin D intakes are somewhat dispersed suggesting a greater variation in vitamin D intake between individuals. The average mean difference in vitamin D intake of the FFQ and three-day FR was 142 IU (285 IU for FFQ and 143 IU for FR). Similarly, a study by Glabaska et al. [32] assessed two FFQs against a three-day FR. Vitamin D intake obtained from FFQ-1 and FFQ-2 were 132 IU ad 144 IU respectively and 76 IU in the three-day FR. Such differences in values lead to the scattering of values in BA plot. The reason for higher vitamin D intakes for FFQ as compared to FR might be that the FFQ is a retrospective method [33]. This allows for participants to recollect and report foods consumed over a specific period of time when compared to the FR, which focus on a limited number of days prospectively. This prospective food intake reporting for a limited number of days does not guarantee that foods containing vitamin D will be consumed within those days [32].

The observed dietary vitamin D intakes in this study’s participants were generally low. The Dietary Reference Intakes and Estimated Average Intakes for vitamin D were 600 IU/day (meets needs of ≈97% of the population) and 400 IU/day (meets needs of ≈50% of the population), respectively [34]. Based on the FFQ intake data, 58 out of 62 subjects had vitamin D intakes <600 IU, indicating a vitamin D insufficiency of ≈94%. This vitamin D insufficiency prevalence by and large matches with the vitamin D insufficiency that was reported for Qatar based on serum 25(OH)D concentrations [35], a widely used biomarker for the assessment of vitamin D nutritional status. This further suggests that the vitamin D-specific FFQ is valid for the assessment of vitamin D intake for Qatari women.

According to the quartile comparison, 45 out of 62 (≈73%) vitamin D intakes from FFQ and FR were in the same quartile or adjacent quartile as the vitamin D intakes from the three-day FR, suggesting a good agreement between vitamin D intakes from the FFQ and the FR. Glabska et al. [36] also reported similar findings. They collected dietary intakes while using two FFQ and compared with the dietary intakes from the three-day FR. The study showed that the first FFQ was highly correlated with the three-day FR as the subjects in the same quartile were 60% (*n* = 45). This was considered to be a high agreement between FFQ and FR. However, the second FFQ only had 37 subjects (49.3%) in the same quartile and the level of agreement is lower than the first FFQ. Another study by Fernandez et al. [37] validated a FFQ against seven-day FR. They estimated the genistein intake and compared while using the quartile classification. They also collected dietary intakes using two FFQs. First, FFQ had 78% of its subject accumulated in the first and second quartile, which suggested high agreement between FFQ and FR. Second, FFQ dietary intakes had a greater level of agreement when compared to the first FFQ in the validation analysis against FR. Approximately 88% subjects were placed in the same quartile and in the adjacent quartile. The differences in the extent of agreement between validation studies is likely due to differences in nutrient intakes by subjects, which is due to the differences in the availability of food sources in that population.

In 2009, Serra-Majem et al. [38] proposed a seven-point system to qualitatively rank dietary intake validation studies based on the European Micronutrient Recommendations Aligned Network of Excellence. This grading system was based on five criteria, i.e., sample size (one point), statistical tests used (three points), data collection procedures (one point), consideration of seasonality (0.5 point), and supplement consumption (1.5 points). A validation study would be graded as very good (>5), good (5–3·5), acceptable/reasonable (3·5–2·5), and poor (<2·5). Based on their grading system, we have achieved a score of 4.0 out of maximum seven points (data analysis 3.0 points and data collection procedure one point). Therefore, qualitatively, our study falls under the “good” category. However, whether this particular grading is applicable for validation of FFQ in countries like Qatar is not known. 

The use of correlation tests in validating nutrient intakes from FFQ against a reference standard is controversial. Lombard et al. [39] reviewed 60 FFQ validation studies in 2015. They reported that out of six statistical tests identified, the correlation (either singly or in conjunction with one or more statistical tests) was the most commonly used test in validation studies. After the correlation test, cross-classification and BA plot tests were the next most commonly used tests. Overall, out of 60 studies that were reviewed by Lombard et al. [39], 57 studies used the correlation test, 28 studies used cross-classification, and 27 studies used the BA plot. In this study, we analyzed the data with these four commonly used tests that were identified by Lombard et al. [39] to validate the vitamin D-specific FFQ against FR. Moreover, the majority of the studies they reviewed, used three or more statistical tests in validating FFQ. Similarly, we also used three tests to validate FFQ against a reference. Although the correlation test is a widely used statistical tool in validation studies, caution should be used in interpreting findings, because this test measures the direction and strength of the relationship between two variables, but not the level of agreement [39,40]. 

In this study, the validation of FFQ against FR was based on dietary intakes that were collected from 62 participants. This sample size by and large matches with the sample size of several studies reported in the literature. For example, Araujo et al. [20] validated FFQ against FR in 66 Brazilian adolescents. Weir et al. [22] used 49 subjects in the validation of FFQ against weighed FR in 18-64 year old Irish. Steinemann et al. [19] used 56 participants in the validation of FFQ against weighed FR in 22–85 year old Germans. For validation studies, a minimum of 50 sample size is recommended [41], although higher is desirable. Thus, we met the criteria for sample size for the validation of FFQ.

A major strength of this study was that we assessed the agreement between FFQ and FR with different tests, i.e., BA plot, cross-quartile classification, and weighted Cohen’s κ. Based on these three tests, we achieved a moderate to good agreement between the vitamin D intakes from FFQ and FR. Another strength of this study was that, in the validation of FFQ tool test, we used nutrition students, because they are familiar with the serving sizes, food ingredients, and procedures used in FFQ and FR. Dietary intakes from FFQ from participants were collected through face-to-face interaction to increase the accuracy of reporting intakes. It is still possible that there might be some reporting error. However, this reporting error may be minimal, given the nature of education background of these participants. In calculating dietary vitamin D intakes, we used the USDA food composition tables for those foods the vitamin D content was not available on the food label. This might have under- or over-estimated the vitamin D intakes from the dietary methods. We did not include vitamin D supplements in the intake analysis, because we wanted to only focus on the vitamin D food sources, as the focus is not the measurement of vitamin D nutritional status. Like many dietary studies that depend on subjects’ memory, this study is also prone for recall bias. However, the recall bias might be limited, because we have used the nutrition students in the validation study, as these students are familiar with FFQ and FR analytical procedures. The subjects in this study were homogeneous and their dietary habits may differ from the dietary habits of Qatar population at large. It is not known how this impacted the validation results. It is also possible that the dietary vitamin D intake from three-day FR might have been underestimated, because subjects might have not consumed vitamin D rich foods within those study validation days. We did not explore the role of over- or under-reporting of energy intake on the analysis. Studies shown that subjects underreport energy intake in FR [42] and over-report energy intake in FFQ [43]. To what extent this underreporting of energy had an impact on the vitamin D intake in this study is not known. Seasonality was not considered in this study, because very little food is produced in Qatar and most of the food is imported into Qatar. Therefore, the effect of the season on the dietary intake of vitamin D might be minimum. We did not take the Sun exposure into account because exposure to Sun in Qatar is limited due to hot conditions and the use of traditional clothing by women. Lastly, although the cross-classification test was the second most commonly used test in validating FFQ, it is prone for chance agreement [39]. It is not known to what extent this bias affected our results.

## 5. Conclusions

In conclusion, an agreement was achieved between the vitamin D intakes of FFQ and three-day FR based on the results from the BA agreement plot (BA index, ≈3.2%) and the quartile comparison test (73% were in the same or adjacent quartile). Overall, the level of agreement is moderate to good. However these findings suggest that the FFQ can be used as a valid dietary method to assess the vitamin D intake in Qatari women. The participants in this study were all women (homogeneous). Although the participants consumed several Qatari foods as well as foods from the West and the South Asian countries, which is a typical food culture in Qatar, the use of only women is a limitation to external validity. Further studies are needed to validate the FFQ for vitamin D intake in Qatari population at large.

## Figures and Tables

**Table 1:** Quartile correlation of vitamin D intake from food frequency questionnaire and three-day food records.

Quartile Difference	Frequency, n	%	Cumulative, n (%)
Classified into same quartile	23	37.10	23 (37.1)
Classified into adjacent quartile (±1)	22	35.48	45 (72.58)
Classified into distant quartiles (2 quartiles apart)	13	20.97	58 (93.55)
Classified into opposite quartiles (misclassified)	4	6.45	62 (100)
Total	62	100	100
